# Effects of Nordic Walking on Prenatal Health: A Focus on Gait Kinematics, Musculoskeletal Pain, and Quality of Life—A Randomized Controlled Trial

**DOI:** 10.3390/healthcare14121788

**Published:** 2026-06-21

**Authors:** Nadia L. Radwan, Olfat Ibrahim Ali, Walaa E. Morsy, Marim Ali M. Slimani, Omkalthoom Sahagi, Sahar Mahmoud Hassan, Zizi M. Ibrahim, Wafaa Mahmoud Amin

**Affiliations:** 1 Department of Biomechanics, Faculty of Physical Therapy, Cairo University, Giza 12613, Egypt; nadiaradwan18@yahoo.com; 2 Department of Health and Rehabilitation Sciences, College of Applied Medical Sciences, Prince Sattam Bin Abdulaziz University, Al-Kharj 11942, Saudi Arabia; 3 Physical Therapy Program, Batterjee Medical College, Jeddah 21442, Saudi Arabia; 4 Department of Basic Science for Physical Therapy, Faculty of Physical Therapy, Cairo University, Giza 12613, Egypt; wafaa_770@yahoo.com; 5 Department of Physical Therapy, College of Nursing and Health Sciences, Jazan University, Jazan 45142, Saudi Arabia; morsy@jazanu.edu.sa (W.E.M.); mariamali@jazanu.edu.sa (M.A.M.S.); usehaqi@jazanu.edu.sa (O.S.); 6 Department of Pediatrics, Faculty of Physical Therapy, Cairo University, Cairo 12613, Egypt; 7 Department of Physical Therapy, Cairo University Hospitals, Cairo 11662, Egypt; 8 Department of Rehabilitation Sciences, College of Health and Rehabilitation Sciences, Princess Nourah bint Abdulrahman University, P.O. Box 84428, Riyadh 11671, Saudi Arabia; zmibrahim@pnu.edu.sa

**Keywords:** Nordic walking, prenatal care, musculoskeletal pain, gait mechanics, quality of life, walking program

## Abstract

**Background/Objectives:** Given the growing need for prenatal care, Nordic Walking (NW) is a promising intervention for maintaining maternal physical activity and quality of life (QoL). We aimed to investigate the influence of NW on gait kinematics, pelvic girdle pain, low back pain (LBP), and QoL during pregnancy. Methods: This is a single-blind randomized controlled trial. A total of 44 pregnant women aged 20 to 40 years with 13–28 weeks of gestation and mild to moderate musculoskeletal pain were included. Participants were randomly assigned to either the study (NW) group or the control group. The study group received the NW program for 12 weeks, three sessions per week, each lasting 45 min. The control group received standard prenatal care plus 30 min of moderate walking three days a week. The GAITRite system was used to measure gait kinematics, and the Visual Analog Scale (VAS) for pain and the SF-36 for QoL were administered at baseline, the fourth week, and the twelfth week. **Results:** NW significantly improved gait kinematics and reduced musculoskeletal pain (*p* < 0.001) with improvements in pain and gait speed exceeding the previous reported MCID thresholds. QoL improved across all SF-36 domains in the NW group (*p* < 0.001) compared with the control group, with large effect sizes observed for the primary outcomes. Conversely, the control group experienced declines in several QoL domains, including energy/fatigue and emotional well-being, despite moderate walking exercise and standard prenatal care over 12 weeks. **Conclusions**: NW may represent an effective prenatal exercise regimen associated with improved gait, reduced pain, and better overall QoL compared with moderate exercise, consistent with standard prenatal care.

## 1. Introduction

Pregnancy is marked by significant physiological, psychological, and biomechanical changes. Studies show that between 50% and 70% of pregnant women experience musculoskeletal pain, which is common in the lower back and pelvic girdle [1,2,3,4]. Effective management strategies are necessary because these conditions significantly impact mobility, everyday activities, and overall QoL.

Hormonal, biomechanical, and vascular changes can contribute to musculoskeletal pain during pregnancy. However, instability and pain in the lower back and pelvis can result from ligament laxity [5]. Additionally, the growing fetus shifts the center of gravity, increasing mechanical pressure on the spine and pelvis and adversely affecting walking patterns [6].

Regular physical activity is strongly advised for better cardiovascular health, a decreased risk of gestational diabetes, and improved mental health during pregnancy [7]. NW uses poles to coordinate upper- and lower-body movement. It increases overall physical demand. The poles help activate the arms, shoulders, chest, and back muscles, thereby increasing total energy expenditure and enhancing posture and balance [8]. This method improves musculoskeletal strength and balance. It also enhances cardiovascular fitness. Tschentscher et al. (2013) claim that NW has significant benefits for QoL, reducing pain and improving physical function across a variety of populations [9]. Studies showed that subjects who practice NW have faster and longer strides [10], with better balance than those who walk frequently or do not exercise at all [11,12].

The effects of NW on the prenatal population have not been adequately studied. The majority of research has concentrated on its effects in older adults [13] or individuals with chronic illnesses [14], with limited focus on pregnancy. This gap in the literature underscores the need for comprehensive research on the effectiveness of NW as a treatment for musculoskeletal discomfort in pregnant women. By evaluating the effects of NW on musculoskeletal pain, gait, and QoL among pregnant women compared with standard prenatal care, this RCT seeks to close this gap.

To ensure an active comparator condition, the control group in this trial also received a prescribed walking program, and standard prenatal care consists of routine obstetric consultations and general advice on physical activity. The study’s findings could influence clinical practice and prenatal care guidelines. This study hypothesized that the NW intervention would significantly improve gait kinematics, reduce musculoskeletal pain, and enhance QoL in pregnant women.

## 2. Materials and Methods

### 2.1. Study Design

An RCT was implemented from 5 November 2024 to 29 June 2025 to investigate the effects of NW on gait, musculoskeletal pain, and QoL in pregnant women. The trial was conducted with a two-group repeated measures design comparing NW training with a control group receiving 30 min of walking three days/week and standard prenatal care. Outcomes were assessed at three time points: baseline, post-learning phase (4 weeks), and the principal phase (12 weeks). The study was conducted in the gynecology, obstetrics, and maternal care departments of hospitals in Al-Kharj City, Riyadh, Saudi Arabia.

### 2.2. Ethical Approval

Participants provided written informed consent. The research adhered to the tenets of the Declaration of Helsinki and was approved by the Research Ethics Committee (RHPT/024/016). The trial was registered on ClinicalTrials.gov under the ID NCT06673147. (URL: https://register.clinicaltrials.gov/prs/beta/studies/S000F2V100000037/recordSummary (accessed on 24 April 2026)). All volunteers had no prior experience with NW and were fully informed about the study’s protocols and potential risks. The authors closely monitored and promptly reported any adverse events. Participants were free to withdraw at any time.

### 2.3. Participants

**Figure 1:**
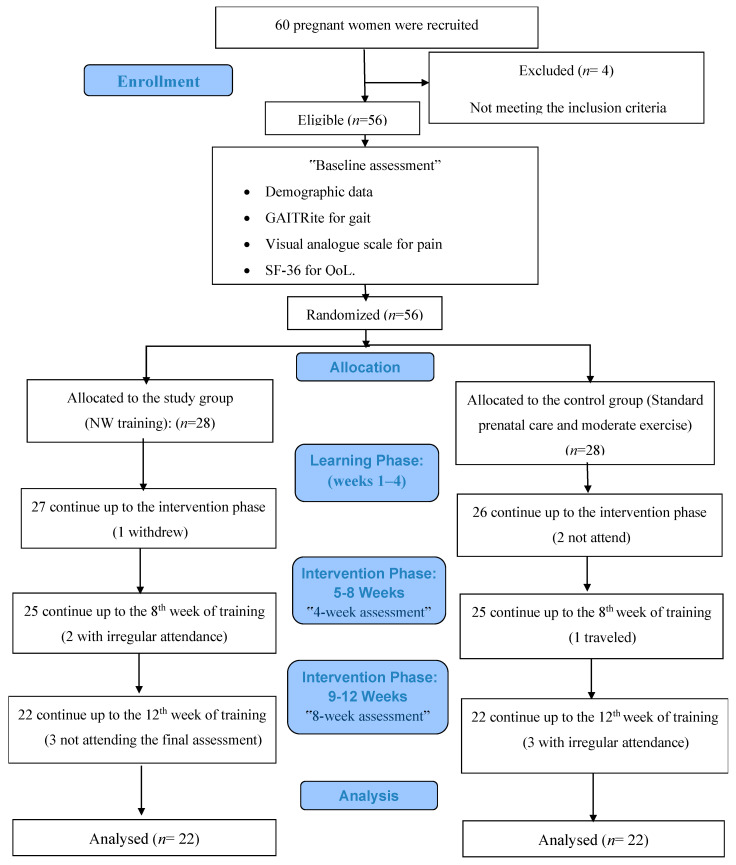
Flowchart.

Recruitment involved healthy women aged 20–40 years with a singleton pregnancy of 13–28 weeks, with mild to moderate musculoskeletal pain, such as lumbar or pelvic girdle pain. Participants were able to perform moderate-intensity physical activity under medical supervision. The period of 13–28 weeks gestation was chosen to maximize the opportunity to observe the presentation of musculoskeletal pain and altered gait following the first trimester and to ensure that the participants could exercise during the period when they tolerate it most easily. This ensured the intervention period finished before the limitations imposed by later pregnancy. Inclusion criteria also included parity (e.g., nulliparous women, primiparous [one previous pregnancy], or multiparous [two or three earlier pregnancies]), an interpregnancy interval of 1 to ≤4 years, and a BMI between 18.5 and 24.9 kg/m^2^ [3,7,15,16]. Exclusion criteria included a history of bony abnormality or lumbar intervertebral disc disease, pain due to non-musculoskeletal pathologies such as urinary tract infections or obstetric complications in pregnancy and high-risk pregnancies, and conditions that restrict physical activity. Women with more than three previous pregnancies or an extremely short (<1 year) or long (>10 years) interpregnancy interval were also excluded [7,16,17,18]. Furthermore, participants who had prior cesarean sections and those who had experience with NW, or were contraindicated from performing exercises during pregnancy, were excluded from the study that fully complied with CONSORT recommendations as presented in the study’s flowchart in Figure 1. Before the study began, adherence was defined as attending at least 80% of the scheduled sessions, a minimum of 29 out of 36 sessions needed to be attended. Those who did not meet this adherence criterion or missed the clinical assessments after the intervention were excluded from the final per-protocol analysis.

### 2.4. Sample Size

Power analysis was performed using G*Power (version 3.1.9.3; Universität Düsseldorf, Düsseldorf, Germany) with a repeated measures ANOVA (within–between interaction) design. Based on an assumed medium effect size (Cohen’s F = 0.3), α = 0.05, and 1–β = 0.80, the required sample size for both groups was 44 (22 in each group). Due to the lack of comparable data for pregnant women, the effect size was derived from earlier research on Nordic Walking interventions conducted with older adults [19].

### 2.5. Randomization

Sixty participants were screened for eligibility; 4 were excluded for not meeting the inclusion criteria. Fifty-six participants were randomized to either the NW or control group using block randomization. To reduce selection bias, sealed, opaque envelopes were used for allocation concealment and were opened sequentially after each subject had been registered. A total of 22 participants in each group completed the intervention and were included in the final analysis. The assessors were blind to group allocation. Still, intervention delivery was separated from measurement evaluation. The number of dropouts and the flow of participants are fully complied with the CONSORT recommendation and graphed in Figure 1.

### 2.6. Outcome Measures

Outcome evaluations were conducted at baseline, after the learning phase (Week 4), and after the main intervention period (Week 12). All evaluations, including gait assessment, musculoskeletal pain measurement, and QoL assessment, were conducted at the same time of day (e.g., in the morning) to minimize variation in participants’ physical and psychological states.

#### 2.6.1. Spatiotemporal Gait Parameters

A GAITRite system was used to assess spatiotemporal gait characteristics on a 61 cm × 5 m walkway with 16,128 pressure sensors. This instrument provides quantitative gait data, which were analyzed using GAITRite GOLD, version 3.2b (CIR System, Inc., USA, Sparta, NJ, 2007). Participants walked a 2-m area in a gymnasium before and after reaching the system, and when leaving it, at their own comfortable speeds. Temporal parameters (gait velocity and cadence) and spatial parameters (stride length, single-limb support%, double-limb support%) were measured three times and analyzed by computer; the average value was used for analysis [20]. According to Kuys et al. (2011), intraclass correlation coefficients (ICCs) for gait speed ranged from 0.96 to 0.98, indicating high test–retest reliability [21].

#### 2.6.2. Musculoskeletal Pain

The visual analogue scale (VAS) is the most used tool for assessing pain intensity, with high sensitivity. The 10 cm horizontal line is divided into 1 cm sections, and each section represents a point on the VAS. As a result, VAS scores range from 0 to 10 points per question. The point on the horizontal line marked by an individual represents the level of pain they experience. The reliability for determining discomfort levels ranges from 0.76 to 0.84 [20].

#### 2.6.3. Quality of Life

The SF-36 Health Survey was used to determine the results. The SF-36 Health Survey is regarded as a valid, reliable, succinct, and general measure of health [22]. The questionnaire included 36 items covering eight domains: physical functioning (PF), role limits caused by physical health problems (RF), bodily pain (BP), general health perceptions (GH), vitality (VT), social functioning (SF), role limits due to emotional problems (RE), and mental health (MH). Scores are linearly transformed so that the highest possible score is 100; higher scores indicate better physical functioning and psychological health. The eight domains can then be further classified into two summary measures: the physical component summary (PCS) and the mental component summary (MCS). These composite scores are converted into norm-based ratings (mean 50; SD 10), with higher scores meaning better physical health and mental well-being [23].

### 2.7. Intervention

The study group received structured NW exercise, while the control group received standard prenatal care combined with a prescribed walking program of 30 min per day, 3 days per week.

#### 2.7.1. Study Group

To maximize walking technique while ensuring safe biomechanical and cardiovascular outcomes, the entire NW program was divided into two phases, adapted from a validated protocol [24]. (1) Learning phase (weeks 1–4): Each week, participants attended three 45-min sessions under supervision. Learning the NW technique at a light-to-moderate intensity (Rate of Perceived Exertion, RPE 11–12) was the main goal. The NW technique was practiced during each session, followed by a warm-up consisting of dynamic stretching and light walking, and a cool-down consisting of static stretching and gentle walking. The safe and correct movement patterns were reinforced at this stage, and the effects of early intervention were measured during the 4-week assessment. (2) Main intervention phase (weeks 5–12): Participants moved on to the 8-week structured NW program following the completion of the learning phase. They engaged in three weekly 45-min supervised sessions at increasing intensities (RPE 13–15). Safe level ground was used for the sessions, and participants were given breaks as needed to ensure their comfort. During this phase, 24 sessions were completed. NW sessions were led by qualified physiotherapists trained in the NW protocol prior to the commencement of the study and experienced in prescribing exercise and rehabilitating musculoskeletal conditions.

Conventional NW poles were held vertically with the elbows flexed to 90 degrees in accordance with the International Nordic Walking Federation’s guidelines. A pole weight of 196 g was selected to standardize training conditions and provide support without excessive upper-limb loading, consistent with previous NW protocols [25]. Height was used to determine the pole length, following the standard Nordic Walking guidelines (participant height (cm) x 0.68), to ensure proper body posture and mechanics [26]. To enable close supervision, group sizes were restricted to ten. Also, to enable safe overload, walking distance and speed gradually changed in response to feedback and performance. This strategy aligns with prenatal exercise guidelines and NW studies [7,9,24].

#### 2.7.2. Control Group

In addition to receiving standard prenatal care, participants were prescribed a moderate exercise regime that included 30 min of walking three days a week. Throughout the entire study, walking was maintained at a comfortable moderate pace. To keep track of their walking time, pace, and any discomfort, they kept a daily walking log. Every week, study staff contacted participants to review logs, provide reminders, and reaffirm compliance. Participants were told to report any negative events immediately and to stop walking if they experienced pain, lightheadedness, or shortness of breath. Every other element of standard prenatal care, such as regular obstetric visits and general lifestyle advice, was unaltered. The American College of Obstetricians and Gynecologists (ACOG) recommend regular physical activity during pregnancy to support the health of both the mother and the fetus. This strategy aligned with their recommendations [27].

Subject adherence was monitored throughout the intervention. For the NW group, adherence was measured by supervised attendance at the sessions. For the control group, adherence was measured using walking logs with weekly follow-up checks on compliance. Safety surveillance was conducted throughout the trial, and no exercise-related adverse events occurred in either group. Adherence of NW subjects was measured by the number of sessions attended during the supervised intervention (36 intended sessions), and for control subjects by the number of walking logs completed. Both groups demonstrated the same completion rate, 78.6% (22/28) for the final analysis, with an attrition rate of 21.4% (6/28) as presented in the CONSORT flow diagram.

### 2.8. Statistical Analysis

According to the Shapiro–Wilk Test, box plots, and histograms, the study outcomes were normally distributed. Homogeneity of variances between groups was assessed using Levene’s test, while homogeneity of variance–covariance matrices were examined using Box’s M test, indicating that this assumption was fulfilled. For repeated-measures effects, Mauchly’s test was used to assess sphericity; when violations were detected, Greenhouse–Geisser corrections were applied. Between-group and within-group differences in the measured outcomes were studied using MANOVA. Effect sizes for repeated-measures MANOVA were reported as partial eta squared (η^2^p). To adjust these overall effect estimates and facilitate interpretation of the magnitude of individual outcomes, Cohen’s d values for between-group comparisons were additionally reported. Post hoc analyses were performed using Bonferroni-adjusted multiple comparisons to control for type I error. A per-protocol analysis was conducted for 44 participants (22 in each group) with ≥80% attendance rate and participants who completed the baseline assessment, the 12-week intervention, and the final clinical assessments.

Demographic characteristics of participants were investigated by an unpaired T-test, and a chi-square test was used for categorical data. Mean and standard deviation were used to represent data. *p* ≤ 0.05 was the significance level, with 95% confidence. IBM ^®^ SPSS ^®^ Statistics Version 25 was used in all statistical analyses.

## 3. Results

### 3.1. Demographic Characteristics of the Participants

An independent t-test revealed no statistically significant differences between groups in age, weight, height, body mass index (BMI), or interpregnancy interval (*p* > 0.05). Moreover, the chi-square test revealed no significant difference between the study and control groups in terms of the number of pregnancies (*p* > 0.05), as shown in Table 1. Regarding intervention fidelity and participant compliance, the final analyzed sample was 22 per group; in the study group, the mean attendance percentage was 85.0%, with an average of 30.6 ± 1.2 completed Nordic walking sessions. In the control group, the mean compliance percentage was 86.1%, with an average of 31.0 ± 1.2 completed exercise sessions.

The multivariate analysis of variance (MANOVA) revealed a statistically significant group by time interaction (F (30,13) = 163.82, *p* < 0.001, η^2^ = 0.997) in addition to group main effect (F (15,28) = 67.75, *p* < 0.001, η^2^ = 0.973), and primary effect of time (F (30,13) = 427.47, *p* < 0.001, η^2^ = 0.999). In interpreting the observed large partial eta squared values, it is essential to consider the repeated-measures design and the relatively consistent within-group responses across time points, as these factors combined with the small sample size may increase the proportion of explained variance. Consequently, outcome-specific Cohen’s d values are also presented in Table 2 and Table 3 to offer a clinically interpretable estimate of treatment effects.

### 3.2. Effect of NW on Pain Level

**Figure 2:**
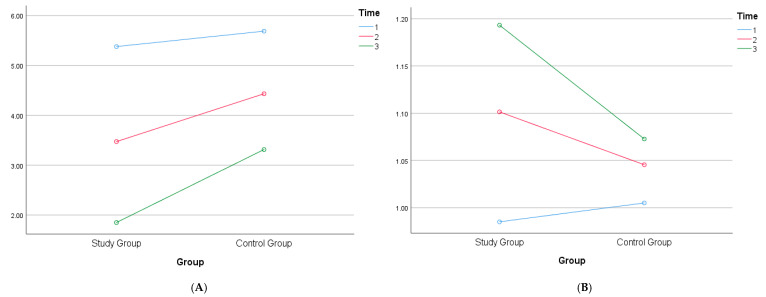
Changes in pelvic pain (**A**) and gait velocity (**B**) from baseline to Week 4 and Week 12.

Within-group comparisons revealed significant decreases in pelvic pain and LBP in both groups at 4 and 12 weeks of intervention (*p* < 0.001). Moreover, between-group comparisons showed no statistically significant difference at baseline (*p* > 0.05). In contrast, significant differences were observed between groups at 4 and 12 weeks of intervention for both pelvic pain and LBP, favoring the study group (*p* < 0.001; Table 2 and Figure 2).

### 3.3. Effect of NW on Gait Kinematics

Table 2 and Figure 2 present the within-group comparison, which revealed that the study group demonstrated significant improvements in gait parameters—velocity, cadence, stride length, double-limb support, and single-limb support—at 4 and 12 weeks of follow-up (*p* < 0.001). Additionally, the control group exhibited significant changes in velocity, cadence, and stride length (*p* < 0.05). However, there were no statistical changes in double limb support and single limb support. Between-group comparisons revealed no significant differences at baseline; however, there were significant improvements in all gait kinematics in the 4 and 12 weeks of follow-up, favoring the study group. (*p* < 0.001).

### 3.4. Effect of NW on QoL

Within-group comparisons showed significant improvements in all QoL domains after 4 and 12 weeks of intervention (*p* < 0.001). In the control group, significant negative changes occurred in physical function after 12 weeks (*p* = 0.021) and role limitations due to emotional problems after 4 weeks (*p* = 0.004); however, no changes were observed in other domains (*p* > 0.05) after either 4 or 12 weeks of follow-up. Between-group comparisons revealed statistically significant changes in all QoL domains after 4 and 12 weeks of follow-up, favoring the study group (*p* < 0.001), as shown in Table 3.

## 4. Discussion

This study is the first randomized controlled trial conducted in Saudi Arabia suggesting that NW may be a safe, practical, and effective exercise regimen associated with decreased musculoskeletal pain and improved gait pattern and QoL in pregnant women. Compared with the conventional prenatal care combined with moderate walking activity in the control group, the organized NW program in the study group resulted in greater improvements in gait kinematics, pain relief, and overall QoL. Pregnant women in both groups continued regular exercise when the programs were supervised and structured, indicating good adherence. The greater achievement by the NW participants indicates that the exercise’s structure and content are essential for producing meaningful clinical outcomes.

### 4.1. Pain Reduction

A pronounced reduction in pelvic and LBP was observed in the NW group compared with the control group. This result is consistent with previous research work demonstrating that organized exercise for pregnant women improves postural control and muscle performance [2,17,28,29]. NW redistributes body weight and activates the trunk and upper-limb muscles. This reduces lumbopelvic load and pain [9,10]. Considering the minimum clinically significant difference (MCID) for pain from previous research (1.4 cm on a 10-cm visual analogue scale) [30], NW participants showed both statistical and clinical significance, as their values exceeded this benchmark; in contrast, most values in the control group were below it.

Our results on musculoskeletal pain align with many systematic reviews that emphasize structured exercise programs for pregnant women, such as Pilates and aquatic therapy, which significantly reduce lumbopelvic pain and enhance functional activities [15,16,31]. Moreover, ACOG supports the use of moderate-intensity aerobic conditioning, strengthening exercises, and postural and core stability training to relieve musculoskeletal pain during pregnancy [18]. Also, in a Cochrane review, researchers have observed that physical activity programs lower the risk and intensity of LBP and pelvic pain without adverse effects [3]. All this evidence supports the potential role of NW as a safe, evidence-informed clinical exercise intervention for pregnant women.

### 4.2. Gait Kinematics and Clinical Significance

Research has confirmed changes in gait kinematics during pregnancy, including decreased stride length and speed, with extended double-support time to maintain balance [32,33,34], findings consistent with our results. In this study, NW was associated with improvements in these kinematic alterations, including increases in stride length, cadence, and speed, and decreases in double-support time, which may contribute to a more efficient gait pattern with less pain. Using poles during NW enhances rhythmic forward propulsion of the body and good trunk stability, thereby building confidence and smoothness in walking [20,24]. Moreover, the observed improvement in gait speed exceeded the recognized MCID of 0.10–0.20 m/s reported by Perera et al. [35], highlighting the potential clinical significance of these findings. However, this MCID was initially determined in an elderly population and has yet to be validated in pregnant women; its relevance to prenatal populations should be approached with caution.

Research suggests that prenatal exercise, including proprioceptive training and postural control, improves gait kinematics and reduces fall risk [36,37]. Improved electromyographic activity in the trunk and hip stabilizer muscles during NW and functional exercises is closely related to gait symmetry and energy efficiency [38,39]. Additionally, gait training with upper-extremity engagement in NW has been associated with reduced mechanical lumbar tension and enhanced trunk stability [40]. Consequently, these findings suggest the potential clinical relevance of NW as a safe locomotor intervention during pregnancy.

### 4.3. Quality of Life

All QoL domains showed significant improvements due to NW. The control group, on the other hand, made only modest and irregular progress. Over the four weeks, role constraints due to emotional issues improved, and by twelve weeks, physical function had also improved. After twelve weeks, minor changes were also observed in energy/fatigue, emotional well-being, pain, and general health. These latter gains were less pronounced and less consistent than those seen with NW, suggesting that the structured program may have a broader impact on QoL, which was more extensive and clinically significant—pregnancy-related pain and mobility issues frequently lower QoL [1]. Additionally, significant declines in QoL were reported in the control group at 12 weeks, with disturbances in energy, emotional well-being, social functioning, general health, and pain. This may be attributed to the increasing physical and psychological demands of advancing pregnancy. The moderate walking program for the control group was likely insufficient to offset the biomechanical stress and fatigue prevalent in later pregnancy. Conversely, the structured nature of the NW program appeared to better support participants’ physical and psychological well-being [1,41,42,43].

These findings are consistent with previous studies showing that health-related QoL commonly deteriorates as pregnancy progresses, particularly during the second and third trimesters, owing to increasing gestational weight, fatigue, musculoskeletal discomfort, and reduced mobility [44]. In contrast, the NW group demonstrated significant improvements across all QoL domains, suggesting that this structured exercise program may help mitigate the decline in QoL commonly observed during later pregnancy.

Increased physical independence and psychological well-being were probably supported by the noted improvements in gait. These results are supported by recent data: organized prenatal exercise increased physical and psychological QoL scores [45,46]. Also, Women who continued to exercise during pregnancy reported greater vitality and fewer role restrictions [47]. These results demonstrate the multifaceted advantages of NW and are consistent with the findings of the current trial.

Meta-analysis studies reported a positive effect of regular practice of supervised prenatal exercise on reducing anxiety and depression, improving sleep quality, and increasing bodily confidence [48,49]. A mind–body exercise regimen, such as yoga and Pilates, significantly reduces fatigue and positively regulates emotions [50]. These closely align with our study results, confirming that NW improves physical function and enhances QoL in a clinically relevant manner.

### 4.4. Clinical Significance and Application

NW is superior to traditional walking because it engages the upper body muscles, increases energy expenditure, and enhances postural support [25]. It is a helpful supplement to prenatal care, particularly for pregnancy-related lumbopelvic discomfort [1]. With 45-min sessions conducted three times a week, NW offers a safe and efficient approach for enhancing gait, reducing musculoskeletal pain, and improving QoL during pregnancy.

### 4.5. Control Group

The control group walked for 30 min three days a week and experienced improvements in gait and discomfort. This may be due to the Hawthorne effect, in which people alter their behavior when they are being watched [51]. Pregnancy-related physiological benefits of moderate walking include improved circulation, reduced pain, and increased well-being [52]. These changes likely fell below the minimum clinically important difference (MCID) thresholds, indicating a limited practical effect.

While it is evident that the NW group experienced the greatest improvement, we must interpret these results cautiously. We cannot claim that the observed improvements are solely attributable to the NW poles or poles-based walking technique alone. Participants in the NW group received more supervised, time-efficient (45 vs. 30 min exercise), therapist-attended sessions and were more closely supervised during exercise and monitored over the course of the intervention compared with the control group. These may have contributed to an increased motivation to participate, better adherence to treatment and increased self-efficacy. We may then conclude that the superiority experienced in the NW group is partly attributed to these non-specific effects and the increased dose of intervention received.

### 4.6. Strengths and Limitations

The trial has several strengths: blinded outcome assessment, a randomized controlled design, and validated measures of pain, gait, and QoL. There were no adverse events, confirming its safety and viability. In contrast to conventional aerobic or resistance training, NW during pregnancy has been the subject of few RCTs. This study demonstrates that pole-assisted walking improves gait mechanics and musculoskeletal pain. NW is an affordable and useful supplement to regular prenatal care. Single-city recruitment, restricted BMI range, lack of participant and instructor blinding, and limited study size are among the limitations. Given the 21.4% attrition rate, the per-protocol approach was used which limits the ability to generalize these findings to patients who struggle with intervention adherence and may overestimate the true clinical treatment effect. Insufficient long-term follow-up prevents evaluation of postpartum persistence. Postpartum outcomes, longer follow-up periods, and a variety of groups should all be included in future research. Moreover, the results may have been affected by differences in exercise duration and supervision levels between groups. Further research is required to use exercise-matched groups with identical exercise durations and supervision levels to eliminate confounding factors.

In addition, the inclusion of pregnancies that are otherwise relatively healthy may reduce the generalizability of the findings to the broader obstetric population, including patients at high risk and with diverse clinical features.

## 5. Conclusions

NW was associated with improvements in gait mechanics, lower musculoskeletal pain, and improved overall QoL among pregnant women compared with conventional prenatal exercise. To maximize maternal physical performance and well-being, our results lend support to the inclusion of structured NW programs in prenatal care interventions.

## Figures and Tables

**Table 1:** Demographic characteristics of the participants.

Variable	Study Group	Control Group	*p*-Value
Age (years)	30.95 ± 5.04	30.79 ± 5.27	0.921
Weight (kg)	54.04 ± 3.86	54.94 ± 4.00	0.454
Height (cm)	155.23 ± 4.28	155.41 ± 3.84	0.883
Pre-pregnancy BMI (kg/m^2^)	22.15 ± 1.64	22.52 ± 1.64	0.461
Number of pregnancies			
1	6 (27.2%)	5 (22.7%)	0.827
2	8 (36.4%)	10 (45.5%)	
3	8 (36.4%)	7 (31.8%)	
Inter-pregnancy interval (years)	1.82 ± 1.24	1.87 ± 1.15	0.880

P: Probability level, significant at *p* ≤ 0.05, n: number, BMI: body mass index, kg: kilogram, cm: centimeter, m^2^: meter^2^.

**Table 2:** Effect of NW on pain and gait kinematics within and between groups.

Variable	SG (M ± SD)	CG (M ± SD)	Within Group Comparison	SG MD (95% CI)	SG P Value	CG MD (95% CI)	CG P Value	Between-Group MD (95% CI)	*p* Value *	Between-Group Effect Size (Cohen’s d)
Pelvic pain										
Baseline pelvic pain	5.38 ± 0.52	5.69 ± 0.54	Baseline vs. 4 Ws	−1.91 (−2.14–−1.68)	<0.001	−1.26 (−1.49–−1.04)	<0.001	−0.31 (−0.20 to −0.42)	<0.001	
Pelvic pain after 4 Ws	3.47 ± 0.42	4.43 ± 0.41	Baseline vs. 12 Ws	−3.53 (−3.76–−3.30)	<0.001	−2.38 (−2.60–−2.15)	<0.001	−0.96 (−0.83 to −1.09)	<0.001	2.31
Pelvic pain after 12 Ws	1.85 ± 0.30	3.31 ± 0.33	4 Ws vs. 12 Ws	−1.62 (−1.85–−1.40)	<0.001	−1.12 (−1.35–−0.90)	<0.001	−1.46 (−1.33 to −1.58)	< 0.001	4.63
LBP										
Baseline LBP	5.29 ± 0.51	5.23 ± 0.44	Baseline vs. 4 Ws	−1.86 (−2.07–−1.65)	<0.001	−1.23 (−1.44–−1.02)	<0.001	0.06 (−0.05 to 0.17)	0.312	
LBP after 4 Ws	3.43 ± 0.38	4.00 ± 0.33	Baseline vs. 12 Ws	−3.03 (−3.24–−2.82)	<0.001	−1.97 (−2.18–−1.76)	<0.001	−0.57 (−0.47 to −0.67)	<0.001	1.6
LBP after 12 Ws	2.26 ± 0.37	3.27 ± 0.35	4 Ws vs. 12 Ws	−1.17 (−1.38–−0.96)	<0.001	−0.74 (−0.95–−0.53)	<0.001	−1.01 (−0.91 to −1.11)	<0.001	2.8
Velocity (m/s)										
Baseline velocity	0.99 ± 0.11	1.01 ± 0.13	Baseline vs. 4 Ws	0.11 (0.08–0.13)	<0.001	0.04 (0.02 to 0.07)	<0.001	−0.02 (−0.05 to 0.01)	0.169	
Velocity after 4 Ws	1.10 ± 0.09	1.05 ± 0.13	Baseline vs. 12 Ws	0.20 (0.17–0.22)	<0.001	0.06 (0.03 to 0.09)	<0.001	0.05 (0.02 to 0.08)	0.003	0.45
Velocity after 12 Ws	1.19 ± 0.09	1.07 ± 0.12	4 Ws vs. 12 Ws	0.09 (0.06–0.12)	<0.001	0.02 (−0.01–0.05)	0.155	0.12 (0.09 to 0.16)	<0.001	1.13
Cadence (steps/min).										
Baseline Cadence	102.55 ± 5.38	103.07 ± 5.40	Baseline vs. 4 Ws	5.44 (4.60- 6.27)	<0.001	0.82 (0.00–1.64)	0.049	−0.52 (−1.42 to 0.39)	0.258	
Cadence after 4 Ws	107.99 ± 4.12	103.89 ± 5.31	Baseline vs. 12 Ws	9.50 (8.66–10.33)	<0.001	0.95 (0.12–1.77)	0.027	4.10 (3.19 to 5.01)	<0.001	0.86
Cadence after 12 Ws	112.05 ± 3.67	104.02 ± 5.38	4 Ws vs. 12 Ws	4.06 (3.23–4.89)	<0.001	0.13 (−0.69–0.96)	0.751	8.03 (7.13 to 8.92)	<0.001	1.74
Stride length (cm)										
Baseline Stride length	117.94 ± 0.96	117.82 ± 1.02	Baseline vs. 4 Ws	2.68 (2.45–2.91)	<0.001	0.21 (−0.02–0.44)	0.074	0.13 (−0.08 to 0.33)	0.22	
Stride length after 4 Ws	120.62 ± 1.09	118.03 ± 1.14	Baseline vs. 12 Ws	5.20 (4.97–5.43)	<0.001	0.28 (0.05–0.51)	0.018	2.59 (2.37 to 2.82)	<0.001	2.32
Stride length after 12 Ws	123.14 ± 1.31	118.10 ± 1.17	4 Ws vs. 12 Ws	2.52 (2.29–2.75)	<0.001	0.07 (−0.16–0.30)	0.551	5.04 (4.80 to 5.28)	<0.001	4.06
Double limb support %										
Baseline Double limb support %	25.53 ± 0.84	24.98 ± 1.08	Baseline vs. 4 Ws	−2.04 (−2.48–−1.60)	<0.001	0.07 (−0.37–0.51)	0.759	0.55 (0.27 to 0.83)	0.064	
Double limb support % after 4 Ws	23.49 ± 1.00	25.05 ± 1.58	Baseline vs.12 Ws	−3.89 (−4.33–−3.45)	<0.001	0.40 (−0.04–0.84)	0.072	−1.56 (−1.83 to −1.30)	<0.001	1.18
Double limb support % after 12 Ws	21.64 ± 1.21	25.38 ± 1.57	4 Ws vs.12 Ws	−1.85 (−2.29–−1.41)	<0.001	0.33 (−0.11–0.77)	0.143	−3.74 (−4.02 to −3.46)	<0.001	2.67
Single limb support %										
Baseline Single limb support %	37.85 ± 0.74	37.51 ± 0.67	Baseline vs. 4 Ws	0.67 (0.43–0.90)	<0.001	−0.08 (−0.31–0.15)	0.505	0.34 (0.19 to 0.49)	0.090	
Single limb support % after 4 Ws	38.52 ± 0.85	37.43 ± 0.61	Baseline vs.12 Ws	1.35 (1.12–1.58)	<0.001	0.07 (−0.16–0.30)	0.556	1.09 (0.90 to 1.29)	<0.001	1.47
Single limb support % after 12 Ws	39.20 ± 0.98	37.58 ± 0.84	4 Ws vs. Ws	0.68 (0.45–0.91)	<0.001	0.15 (−0.08–0.38)	0.198	1.62 (1.40 to 1.85)	<0.001	1.77

P: Probability level, * significant at *p* ≤ 0.05, *p* value *: significance level between groups, Ws: weeks, SG: Study Group, CG: Control Group, %: percentage, M ± SD: Mean ± standard deviation. MD (95% CI): Mean difference (95% confidence interval), vs: versus.

**Table 3:** Effect of NW on the QoL within and between groups.

Variable	SG (Mean ± SD)	CG (Mean ± SD)	Within-Group Comparison	SG MD (95% CI)	SG P Value	CG MD (95% CI)	CG P Value	Between-Group MD (95% CI)	*p* Value *	Between-Group Effect Size (Cohen’s d)
Physical function										
Baseline Physical Function	32.27 ± 14.03	37.50 ± 13.25	Baseline vs. 4	−18.64 (−25.26–−12.01)	<0.001	−2.05 (−8.67–4.57)	0.537	−5.23 (−11.00–0.55)	0.077	
Physical function after 4 Ws	46.59 ± 9.18	35.45 ± 10.90	Baseline vs. 12	−18.77 (−26.04–−11.50)	<0.001	−7.95 (−14.65–−1.25)	0.021	−21.82 (−28.98–−14.66)	<0.001	1.1
Physical function after 12 Ws	58.86 ± 8.30	29.55 ± 10.79	4 vs. 12	−0.14 (−6.95–6.67)	0.967	−5.91 (−12.39 −0.57)	0.071	−16.05 (−23.90–−8.20)	<0.001	3.05
Role limitations due to physical health										
Baseline Role limitations due to physical health	13.64 ± 14.90	18.18 ± 17.56	Baseline vs. 4	23.86 (17.58–30.13)	<0.001	2.27 (−4.01–8.56)	0.467	−4.55 (−12.99–3.90)	0.285	
Role limitations due to physical health after 4 Ws	37.50 ± 14.94	20.45 ± 12.53	Baseline vs. 12	46.59 (40.38–52.80)	<0.001	−14.77 (−21.13–−8.42)	<0.001	17.05 (10.59–23.50)	<0.001	1.24
Role limitations due to physical health after 12 Ws	60.23 ± 12.58	3.41 ± 8.78	4 vs. 12	22.73 (16.63–28.82)	<0.001	−17.05 (−23.49–−10.61)	<0.001	56.82 (50.84–62.80)	<0.001	5.28
Role limitations due to emotional problems										
Baseline Role limitations due to emotional problems	13.50 ± 16.61	19.50 ± 16.61	Baseline vs. 4	33.41 (25.43–41.39)	<0.001	12.09 (4.11–20.08)	0.004	−6.00 (−13.91–1.91)	0.133	
Role limitations due to emotional problems after 4 Ws	46.91 ± 17.11	31.59 ± 16.24	Baseline vs. 12	67.00 (59.41–74.59)	<0.001	−1.50 (−9.62–6.62)	0.707	15.32 (8.02–22.62)	<0.001	0.92
Role limitations due to emotional problems after 12 Ws	80.50 ± 16.61	18.00 ± 16.82	4 vs. 12	33.59 (25.80–41.37)	<0.001	−13.59 (−21.46–−5.73)	0.001	62.50 (54.34–70.66)	<0.001	3.74
Energy/Fatigue										
Baseline Energy/Fatigue	37.95 ± 10.76	37.50 ± 9.61	Baseline vs. 4	10.45 (6.81–14.10)	<0.001	−1.59 (−5.20–2.02)	0.38	0.46 (−4.43–5.34)	0.852	
Energy/Fatigue after 4 Ws	48.41 ± 8.36	35.91 ± 7.18	Baseline vs. 12	23.18 (19.51–26.85)	<0.001	−7.73 (−11.45–−4.01)	<0.001	12.50 (8.84–16.16)	<0.001	1.6
Energy/Fatigue after 12 Ws	61.14 ± 6.89	29.77 ± 8.38	4 vs. 12	12.73 (9.06–16.40)	<0.001	−6.14 (−9.86–−2.43)	0.002	31.36 (27.69–35.03)	<0.001	4.09
Emotional well-being										
Baseline Emotional Well-being	41.45 ± 8.60	38.91 ± 9.33	Baseline vs. 4	10.73 (7.39–14.07)	<0.001	−1.27 (−4.61–2.07)	0.448	2.55 (−1.56–6.65)	0.222	
Emotional well-being after 4 Ws	52.18 ± 7.85	37.64 ± 7.47	Baseline vs. 12	24.18 (20.84–27.52)	<0.001	−6.27 (−9.61–−2.93)	<0.001	14.55 (11.14–17.96)	<0.001	1.9
Emotional well-being after 12 Ws	65.64 ± 6.72	32.64 ± 7.17	4 vs. 12	13.45 (10.11–16.79)	<0.001	−5.00 (−8.34–−1.66)	0.004	33.00 (29.85–36.15)	< 0.001	4.75
Social functioning										
Baseline Social functioning	35.45 ± 9.22	38.86 ± 10.88	Baseline vs. 4	16.45 (12.87–20.04)	<0.001	−3.32 (−6.91–0.27)	0.068	−3.41 (−8.12–1.30)	0.15	
Social functioning after 4 Ws	51.91 ± 8.06	35.55 ± 7.46	Baseline vs. 12	31.95 (28.31–35.59)	<0.001	−9.50 (−13.09–−5.91)	<0.001	16.36 (12.83–19.90)	<0.001	2.11
Social functioning after 12 Ws	67.41 ± 8.26	29.36 ± 6.13	4 vs. 12	15.50 (11.92–19.09)	<0.001	−6.18 (−9.77–−2.60)	0.001	38.05 (34.62–41.47)	<0.001	5.23
General Health										
Baseline General Health	36.36 ± 8.48	40.68 ± 10.04	Baseline vs. 4	0.00 (−3.07–3.07)	1	−2.73 (−5.81–0.36)	0.081	−4.32 (−8.68–0.04)	0.052	
General Health after 4 Ws	36.36 ± 8.48	37.95 ± 7.51	Baseline vs. 12	23.41 (20.40–26.42)	<0.001	−8.86 (−11.87–−5.85)	<0.001	−1.59 (−5.20–2.02)	0.38	0.2
General Health after 12 Ws	59.77 ± 6.45	31.82 ± 6.82	4 vs. 12	23.41 (20.39–26.42)	<0.001	−6.14 (−9.15–−3.13)	<0.001	27.96 (25.11–30.80)	<0.001	4.21
Pain										
Baseline pain	35.50 ± 11.01	39.41 ± 9.55	Baseline vs. 4	14.55 (10.37–18.72)	<0.001	−4.05 (−8.22–0.13)	0.057	−3.91 (−8.76–0.94)	0.111	
Pain after 4 Ws	50.05 ± 10.49	35.36 ± 7.75	Baseline vs. 12	26.32 (22.14–30.49)	<0.001	−10.32 (−14.49–−6.15)	<0.001	14.68 (10.46–18.91)	<0.001	1.6
Pain after 12 Ws	61.82 ± 8.13	29.09 ± 6.22	4 vs. 12	11.77 (7.59–15.94)	<0.001	−6.27 (−10.44–−2.10)	0.003	32.73 (29.07–36.38)	<0.001	4.52

P: Probability level, * significant at *p* ≤ 0.05, *p* value *: significance level between groups, Ws: weeks, SG: Study Group, CG: Control Group, %: percentage, M ± SD: Mean ± standard deviation. MD (95% CI): Mean difference (95% confidence interval), vs: versus.

## Data Availability

The data supporting this study’s findings are available on reasonable request. The dataset cannot be made publicly available or shared in anonymized form due to ethical and confidentiality constraints.

## Abbreviations

The following abbreviations are used in this manuscript:

QoL	Quality of Life
LBP	Low back pain
NW	Nordic walking
MCID	Minimal clinical important difference
VAS	Visual analogue scale
